# Development and evaluation of a novel contamination device that targets multiple life-stages of *Aedes aegypti*

**DOI:** 10.1186/1756-3305-7-200

**Published:** 2014-04-25

**Authors:** Janneke Snetselaar, Rob Andriessen, Remco A Suer, Anne J Osinga, Bart GJ Knols, Marit Farenhorst

**Affiliations:** 1 In2Care BV, Costerweg 5, Wageningen 6702 AA, The Netherlands

**Keywords:** *Aedes aegypti*, Dengue, Ovitraps, Vector control, *Beauveria bassiana*, Pyriproxyfen

## Abstract

**Background:**

The increasing global threat of Dengue demands new and easily applicable vector control methods. Ovitraps provide a low-tech and inexpensive means to combat Dengue vectors. Here we describe the development and optimization process of a novel contamination device that targets multiple life-stages of the *Aedes aegypti* mosquito. Special focus is directed to the diverse array of control agents deployed in this trap, covering adulticidal, larvicidal and autodissemination impacts.

**Methods:**

Different trap prototypes and their parts are described, including a floater to contaminate alighting gravid mosquitoes. The attractiveness of the trap, different odor lures and floater design were studied using fluorescent powder adhering to mosquito legs and via choice tests. We demonstrate the mosquitocidal impacts of the control agents: a combination of the larvicide pyriproxyfen and the adulticidal fungus *Beauveria bassiana*. The impact of pyriproxyfen was determined in free-flight dissemination experiments. The effect on larval development inside the trap and in surrounding breeding sites was measured, as well as survival impacts on recaptured adults.

**Results:**

The developmental process resulted in a design that consists of a black 3 Liter water-filled container with a ring-shaped floater supporting vertically placed gauze dusted with the control agents. On average, 90% of the mosquitoes in the fluorescence experiments made contact with the gauze on the floater. Studies on attractants indicated that a yeast-containing tablet was the most attractive odor lure. Furthermore, the fungus *Beauveria bassiana* was able to significantly increase mortality of the free-flying adults compared to controls. Dissemination of pyriproxyfen led to >90% larval mortality in alternative breeding sites and 100% larval mortality in the trap itself, against a control mortality of around 5%.

**Conclusion:**

This ovitrap is a promising new tool in the battle against Dengue. It has proven to be attractive to *Aedes aegypti* mosquitoes and effective in contaminating these with *Beauveria bassiana*. Furthermore, we show that the larvicide pyriproxyfen is successfully disseminated to breeding sites close to the trap. Its low production and operating costs enable large scale deployment in Dengue-affected locations.

## Background

Globally, 2.5 billion people are at risk of becoming infected with Dengue fever [1], a mosquito-borne disease for which there is no specific medication or vaccine. With over 390 million cases annually [2], Dengue is currently the fastest spreading infectious disease in the tropics. Costs to contain the disease are huge and put severe pressure on (health) budgets of affected countries. Without drugs or a vaccine, control of mosquitoes that transmit the virus remains the sole option to control the disease. Contemporary mosquito control focuses primarily on larval source management in the form of breeding site removal or larviciding and adult control through fogging with insecticides [3].

The main vector of Dengue is the yellow fever mosquito *Aedes aegypti* (L.), a diurnal species that displays skip-oviposition behavior (i.e. lays small numbers of eggs in multiple sites [4]) and prefers man-made containers as oviposition sites [5]. These sites are often small and difficult to locate, which makes effective larviciding difficult. The preference of *Aedes* mosquitoes for container-like breeding sites provides the opportunity to control gravid mosquitoes using ovitraps. An ovitrap basically consists of a black or dark colored container filled with water with one or several attractants to lure mosquitoes. Egg-laying female mosquitoes are attracted to the trap by the water [6], visual cues [7], natural odors (mostly from plant infusions) [8-11], conspecifics [12], or synthetic odors [5,13-15]. Ovitraps have an advantage over other traps (for host-seeking mosquitoes) because they do not require a power source or additional carbon dioxide and are not dependent on trap operator’s skill and motivation.

Over the years, various ovitraps have been developed and tested against *Aedes* mosquitoes. Originally, ovitraps were designed as ‘egg dump’ devices [6], killing all larvae hatching inside the trap. However, since *Aedes* females show skip-oviposition behavior this targets only a minor proportion of the lifetime reproductive output by females. Novel ovitraps were therefore designed to also target the adult mosquito. These traps include designs such as the ‘sticky’ trap [7,16,17] or ‘double-sticky’ trap [8] in which gravid mosquitoes are captured using glue, or lethal ovitraps [18-20] in which mosquitoes are exposed to insecticides. A major disadvantage of these lethal ovitraps is the fact that insecticides deployed in such traps have shown reduced efficacy due to widespread insecticide resistance in *Aedes* populations [21].

There are promising alternative mosquito control agents that have been proposed for use in ovitraps, notably autodissemination agents, which are larvicidal compounds that are dispersed to breeding sites by contaminated adult female mosquitoes. Pyriproxyfen is a WHO-recommended juvenile hormone analogue that targets mosquito larvae at the pupal development stage and can be effective in extremely low concentrations (<1 ppb) [22]. It is already being deployed as a mosquito larvicide and is approved for use in drinking water in low concentrations. Experiments have shown that female mosquitoes can acquire pyriproxyfen crystals when landing on a treated surface and deposit these in breeding sites they subsequently visit [23,24], hence killing their offspring and other larvae already present in those breeding sites at the time when these pupate. Because *Aedes* mosquitoes are skip-ovipositors, pyriproxyfen can be used as an autodissemination agent for ‘mosquito-driven larval control’; utilizing the gravid female to disperse the larvicide and contaminate multiple breeding sites in the vicinity [25,26].

Field studies with pyriproxyfen have shown good potential for this new type of vector control [26]. Considering that the contaminated mosquito loses the pyriproxyfen crystals from her legs over time [23], it would be advantageous to deploy this agent in such way that the timeframe between pick up and transfer is as short as possible by contaminating gravid females that lay their first batch of eggs. This would be possible via an ovitrap that contaminates the adult mosquito with pyriproxyfen and allows her to leave the trap afterwards. Considering that *Aedes* mosquitoes typically only need a short time-frame to (skip) oviposit, the addition of a slow-killing adulticide to target the contaminated adult would increase the control impact of such a device. Slow-killing biopesticides, such as entomopathogenic fungi, would be suitable candidates for this purpose. Spores of the fungus *Beauveria bassiana* have been shown to effectively infect mosquitoes upon contact by penetrating the insect cuticle and growing into the haemocoel [27]. This infection reduces the mosquito’s vectorial capacity [28,29], inhibits Dengue virus replication inside the mosquito [30] and eventually kills the mosquito*.* An additional benefit of this fungus is that it is highly virulent to insecticide-resistant mosquitoes [27,31] and even has the potential to augment the efficacy of chemical insecticides [27,32]. The relatively slow kill and pre-lethal impacts of *B. bassiana* can prevent Dengue transmission and at the same time enable effective dissemination of pyriproxyfen by contaminated mosquitoes to surrounding breeding sites.

Whereas contemporary ovitraps have shown good potential in reducing the number of *Ae. aegypti* in an area when deployed in sufficiently high numbers [5], they are mainly used for scientific and monitoring purposes and not commonly deployed as a standard *Aedes* control tool. This opens the opportunity for a trap that can be manufactured on a large scale for the pest control market.

Here we describe the development of a new type of ovitrap, a multi-impact contamination device for *Aedes* mosquitoes. Our aim was to create a user-friendly control device that does not rely on electricity or chemical insecticides. We show the steps taken to design a trap that is attractive to egg-laying *Ae. aegypti* and meets requirements for large-scale manufacturing. Experiments were performed to optimize device attractiveness, including tests with several odor lures to augment attraction to *Aedes* mosquitoes. We show how the device design and the deployment of a new type of gauze enables effective contamination of ovipositing *Aedes* females. In the second part of this paper we demonstrate the potential adulticidal, autodissemination and larvicidal impacts of the agents deployed in the trap. We report for the first time the combination of the control agents *B. bassiana* and pyriproxyfen. Experiments were set up to demonstrate the impact of this mixture, including measurements of lethal impacts on contaminated adults, larvicidal impacts inside the trap and larvicidal impacts in surrounding breeding sites.

## Methods

### Mosquito rearing

Experiments were conducted using laboratory reared *Ae. aegypti* mosquitoes. This colony originates from adults collected in the Caribbean (Aruba) in 2011. Mosquitoes were reared at a temperature of 27(±1)°C and a relative humidity of 65(±5)%. Mosquitoes had an artificial light-dark cycle of 12/12 h (L:D). Mosquito larvae were reared on tap water and fed daily on Tetramin® water tablets for bottom dwelling fish (Melle, Germany). Adult mosquitoes were kept in 30×30×30 cm gauze Bugdorm® cages and had *ad libitum* access to a 6% glucose solution on filter paper. Mosquitoes were fed on human blood twice a week, either through direct feeding on the arm of a volunteer or on membranes.

Trap validation tests utilized gravid females (7-8 days old) at the time they were ready to lay eggs. Prior to the experiments, blood-fed females were selected manually with a mouth aspirator and placed in a container with access to a 6% glucose solution on cotton wool. These females were then kept for 4 days to become gravid, before being used in experiments.

### Evaluation of oviposition attractants

To augment the attractiveness of the trap, several odor lures for gravid *Ae. aegypti* were evaluated (Table 1). We tested a commercially available synthetic mosquito odor lure tablet (AtrAedes), which contains odors identified from volatile grass infusions (*Panicum maximum*, Jacq.) and has been used in other oviposition traps designed to lure gravid *Aedes aegypti* mosquitoes [5,13,14]. Teabags, organic water (from a local ditch) and oak leaf (*Quercus* spp.) infusions were selected because it was shown that fermenting solutions of organic matter are attractive to *Aedes* mosquitoes [9,33]. We also tested alfalfa tablets, which were previously used in ovitraps as an odor lure [10]. We selected tablets with yeast as the main ingredient as an attractant because of its carbon dioxide production and enhancement of bacterial growth in water, and tea capsules as an easily available organic substance with similar characteristics to yeast.

**Table 1:** Selected odor lures and method of preparation for experiments

**Attractant**	**Preparation**
AtrAedes tablets	1 AtrAedes tablet placed in the water of the trap
Oak leaf infusion	Several oak leaves were placed in a bucket with 8 L of boiling water; then left for 2-4 weeks
Earl grey tea	3 teabags were placed in a bucket with 8 L of boiling water
Organic water	Water collected from a local ditch and stored in a bucket for 2 weeks
Alfalfa (tablets)	4 tablets were placed in a bucket with 8 L water and stored for 4 days
Yeast (tablets)	4 tablets were placed in a bucket with 8 L water and stored for 4 days
Green tea (capsules)	4 capsules were placed in a bucket with 8 L water and stored for 4 days

**Figure 1:**
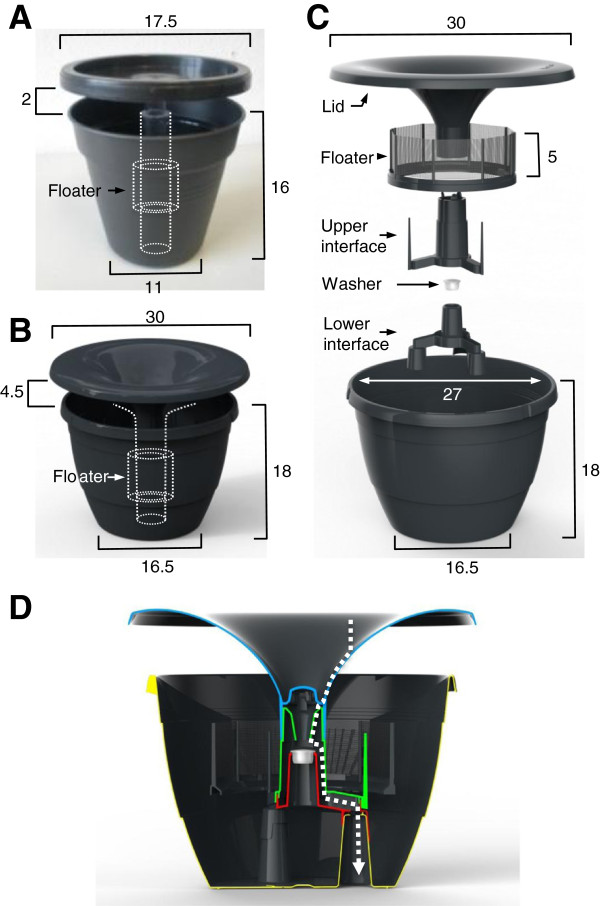
**In2Care® Mosquito Trap design. A** and **B**: Tested prototypes and position of the central tube and floater (dimensions in cm). **C**: Final design of the In2Care® Mosquito Trap. **D**: Cross section of the trap showing water flow (white arrow) with use of the washer. (Rain) water enters through the lid (blue), into the upper interface (green). The washer prevents the water from filling up the trap so that it leaves the trap trough the lower interface (red) and the drainage openings (yellow).

**Figure 2:**
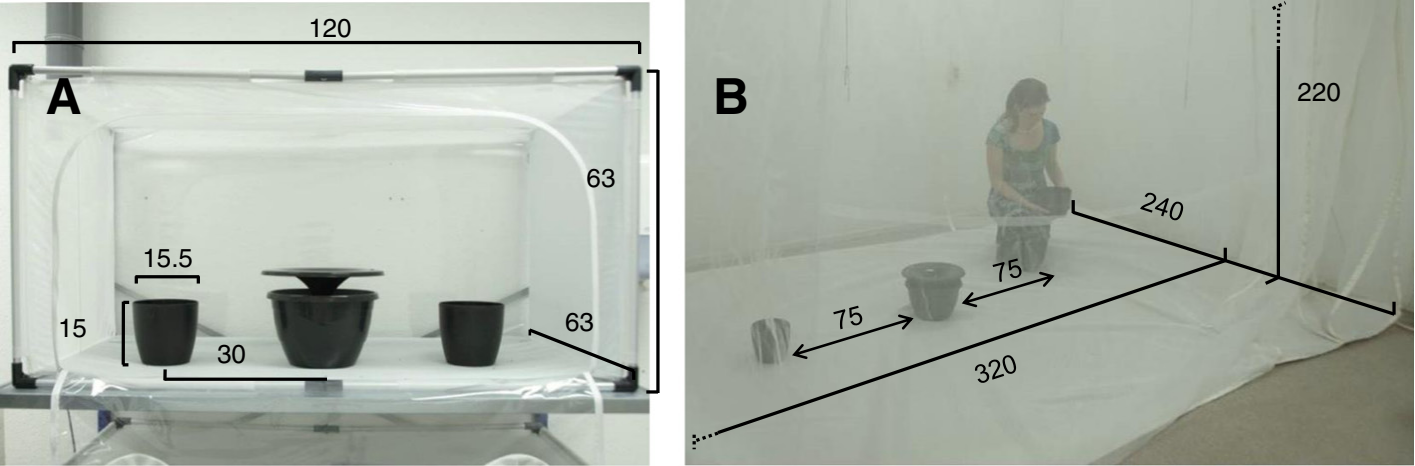
**Experimental cages (dimensions in cm) and position of traps and containers. A**: Free-flight cage used for the experiments with mosquitocidal agents. **B**: Free-flight cage used for the evaluation of oviposition attractants and floater designs. All cages were kept under similar climate conditions (27(± 1)°C, 65(± 5)% RH).

Comparisons between tap water and these attractants were undertaken with trap prototype A (Figure 1A) in a free-flying cage (Howitec Netting BV, Bolsward, The Netherlands, Figure 2B). To measure mosquito attraction we added 200 μl of Aquatain (Aquatain Products Pty Ltd, Kyneton, Victoria, Australia) to 300 ml of the odor-baited water sample and to the control. Aquatain™ (polydimethylsiloxane 78% w/v) is a liquid that creates a monomolecular film on water, which lowers the water surface tension. This silicone oil film has been shown to cause female mosquitoes to drown whilst ovipositing [34], meaning that it can be used to determine the first choice of oviposition location for *Ae. aegypti*.

The trap containing the odor bait was placed on one side of the cage and a trap filled with tap water was placed on the opposite side of the cage. For each comparison fifty gravid *Ae. aegypti* (7-8 days old; four replicates) were released in the cage and positions were switched between the replicates to minimize position effects. After 3 days the number of drowned mosquitoes in each trap was counted to determine their preferred oviposition site. For the comparison between yeast tablets, alfalfa tablets and green tea capsules 3 traps were placed in a row, and the same experimental procedure was followed.

### Optimizing floater design

The floater was tested and improved using trap prototype B (Figure 1B). The trap was placed in the center of the cage (Figure 2B) and filled with 2 L water and a yeast tablet. The floaters supported black polyester gauze (Van Heek BV, Losser, The Netherlands) that was dusted with fluorescent powder (BVDA International BV, Haarlem, the Netherlands) and carefully placed on the water surface in the center of the trap. Two alternative breeding sites were positioned in the cage at opposite sides of the trap to provide competitive breeding sites. These consisted of a black pot in which a transparent plastic container with tap water (500 mL) was placed. For Floater-I and Floater-II 3 replicates were conducted, for Floater-III, Floater-IV and Floater-V 4 replicates were conducted. Fifty mosquitoes were allowed to oviposit for 2 days, after which they were recaptured using a mouth aspirator. These were killed in the freezer and the presence of fluorescent powder on the legs and body determined using a UV light microscope. The proportion of mosquitoes with fluorescent dye was used as a proxy for trap visitation and contact with the gauze on the floater.

### Control agents

We used *Beauveria bassiana* spores from the GHA strain (Laverlam international corporation, Butte, USA), which were produced through solid-state fermentation. Dried spores were kept at low humidity at 5°C until use. Pyriproxyfen (Chemos GmbH, Regenstauf, Germany) was mixed with fungal spores and inert dust particles to create a dust mixture suitable for application on the gauze. This mixture was used inside the final design of the trap (Figure 1C). The powders were applied to the gauze by shaking it in a container with an excess amount of the powder mixture. We deployed 5 × 55 cm strips of gauze dusted with the mixture, which were subsequently fixed around the pins of the floater (Table 2, Type-V). One trap and 2 alternative breeding sites, each containing water with 20 *Aedes* larvae (stage L4) and Tetramin® fish food were placed in a free-flying cage (MegaView Science, Taiwan, Figure 2A). Two plastic cups with tap water, 20 larvae and fish food, were placed outside the experimental cage as a control treatment to measure adult emergence. For each experiment, 50 free-flying gravid *Aedes* females were allowed to oviposit for 2 days, after which they were recaptured. To measure adulticidal impacts of the fungus, 8 control replicates and 8 replicates with *B. bassiana* were conducted and adult survival was monitored for 18 days. Pyriproxyfen dissemination was tested by measuring larval development (% adult emergence) in the two containers next to the trap and compared to adult emergence from control containers, 4 replicates were conducted for the pyriproxyfen tests.

**Table 2:** Measurement of floaters and position of gauze on the floater, as tested in the floater optimization tests

**Floater type**	Outer ø* in cm	Inner ø* in cm	**Height in cm**	**Position of gauze**
Floater-I (small)	5,5	3,5	6,5	Outer ring of floater covered with gauze
Floater-II (medium)	10,0	3,5	5,0	Outer ring of floater covered with gauze
Floater-III (large)	16,0	3,5	5,0	Outer ring of floater covered with gauze
Floater-IV (ring)	16,0	12,0	5,0	Outer and inner ring of floater covered with gauze
Floater-V (pins)	16,0	14,0	1,5	Gauze on top of floater, stabilized with pins

*Ø = diameter.

### Statistical analyses

Statistical analyses were done using SPSS 21.0 software. Normality of the data was investigated using the Shapiro-Wilk Test, a Log_10_ transformation was used if data was not normally distributed. Homogeneity of variances was tested with Levene’s Test (untransformed data). Comparisons between oviposition attractants were done using independent sample T-tests for normally distributed data and a Mann-Whitney U test for data that were not normally distributed. The comparison between tea capsules, alfalfa tablets and yeast tablets was performed using a one-way ANOVA test followed by a Tukey post-hoc test. Analyses of the floater optimizations and the autodissemination impact were done using a one-way ANOVA test followed by a Tukey post-hoc test. The impact of *B. bassiana* on adult mosquitoes was analyzed with a Kaplan-Meier model followed by analysis with the logrank test. LT_50_ data (median lethal time) was obtained from the survival analysis. Replicates were pooled for both controls and fungus groups. Survival curves of infected groups were compared to control groups.

## Results

### Device design and optimization

Trap design development started with a simple flowerpot (Figure 1A, prototype A), comprising a black water container, a central tube and a black lid. The black container provides a visually attractive and sheltered breeding site for Dengue mosquitoes and is commonly used for ovitraps [7,15]. Over time, the volume of the container was increased so that it could contain 3 L of water maximum, which allowed for longer trap use and less frequent maintenance (Figure 1B, prototype B). The final design, a stackable pot with 3 water drainage openings in the bottom, is based on a mass-produced, and therefore low-cost, flower pot (Epla Nora-pot, Desch PlantPak, the Netherlands). The inside of the container is made from smooth, polished polyethylene, to discourage egg-laying mosquitoes to rest on this surface, thereby increasing the chance that they will land on the gauze treated with control agents.

We developed a removable lid to protect the control agents against direct sunlight and rainwater, to allow access for maintenance purposes, and to prevent direct contact between children and/or pets and the bioactives on the gauze. In later development stages (prototype B), the diameter of the lid was increased with a slight overhang to provide better protection against heavy rain. Our tests showed that mosquitoes have a preference for an entry opening between the lid and pot of 4-6 cm (data not shown).

The shape of the original flat-top lid (prototype A) was adjusted to prevent the accumulation of stagnant water on top of the lid, which could potentially form a mosquito breeding site. We added a central lid opening that allows replenishment with water and optional collection of rainwater via a central hollow tube. The tube has a valve through which (rain) water flows into the trap container, avoiding a low water level due to evaporation. Simultaneously, when the water level rises above a certain point, excess water will flow out of the trap through this valve via the central tube, which connects to the pot drainage openings in the bottom. The final design (Figure 1C, the ‘In2Care® Mosquito Trap’) contains a click-on interface that is put on top of the drainage openings, and has 3 protruding extensions that stabilize the floater component.

The floater component was designed to carry vertically placed gauze with mosquitocidal agents and is one of the important novelties of this ovitrap compared to other designs. The In2Care® Mosquito Trap deploys a floater that remains level with the water surface and provides an attractive landing and resting surface for ovipositing mosquitoes. The floater can carry gauze or other materials that are treated or impregnated with mosquitocidal agents and keeps these dry even when water levels fluctuate inside the device.

### Evaluation of oviposition attractants

**Figure 3:**
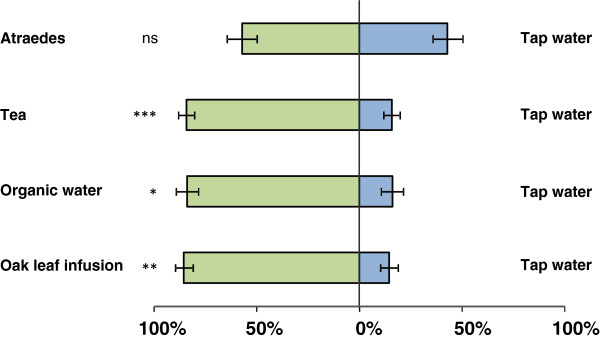
**Dual-choice (% ± SE) experiments with different attractants for gravid *Ae. aegypti.*** Fifty gravid mosquitoes were released per replicate (4 replicates for each comparison). * P < 0.05, ** P < 0.01, *** P < 0.001.

Attractant tests showed an increase in attractiveness of the device when using oak leaf infusions, teabags or organic water (Figure 3)*.* Traps containing these lures were able to catch (i.e. drown due to the presence of Aquatain™) on average more than 80% of all released (free-flying) gravid mosquitoes. Significantly more females drowned in the devices with tea, organic water and oak leaf infusions compared to clean tap water (p < 0.001, p = 0.029 and p = 0.005, respectively). This demonstrates that these organic odors are attractive to gravid *Ae. aegypti*. AtrAedes tablets, however, did not show an effect when used in our setup (p = 0.273). Because the AtrAedes tablets did not increase trap attractiveness, we selected other commercially available tablets for further tests. We examined the most attractive odor (tea) and looked for commercially available tablets or capsules.

**Figure 4:**
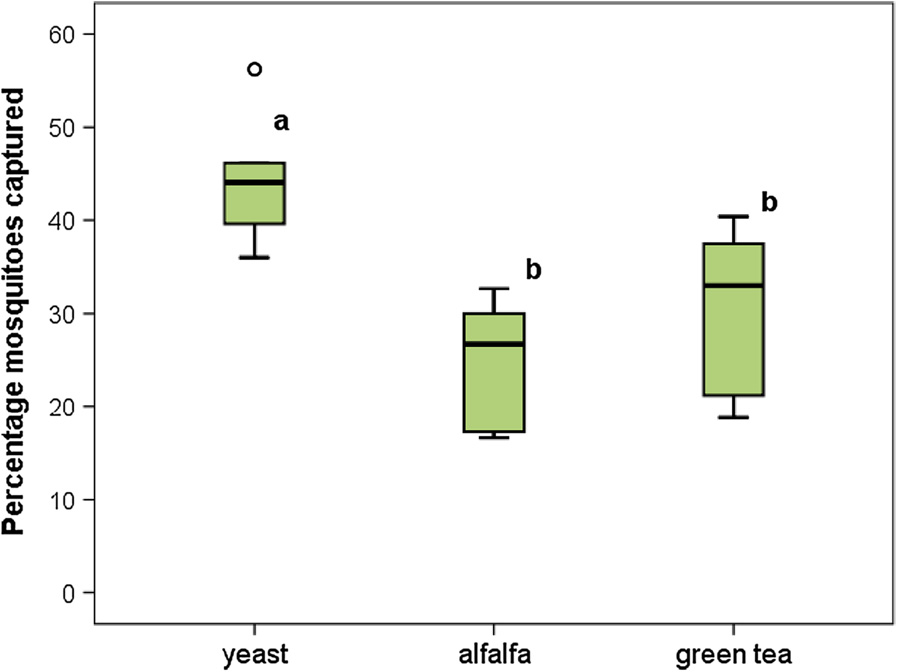
**Percentage (± SE) of mosquitoes collected in oviposition traps baited with different odors.** Fifty gravid mosquitoes were released per replicate (n = 4). Comparisons were made between the 3 odor-baited traps. Each box denotes the median as a line across the middle and the quartiles (25th and 75th percentiles) at the bottom and top. Treatments without letters in common are significant different at P < 0.05. ‘°’ denotes an outlier.

We compared the attractiveness of tea capsules to two other ready-to-use attractants, namely yeast tablets and alfalfa tablets. Yeast was found to be significantly more attractive when deployed in the ovitrap compared to alfalfa (Figure 4, p = 0.001) and green tea (p = 0.018). On average, 45% of the free-flying mosquitoes selected the yeast-baited trap as their first oviposition site compared to the other two odor lures. No significant difference was found between alfalfa and green tea tablets.

Because yeast tablets were significantly more attractive compared to the tested other odor lures and because the tablets are commercially available, we selected the yeast tablets as the standard oviposition attractant in the trap.

### Floater optimization

**Figure 5:**
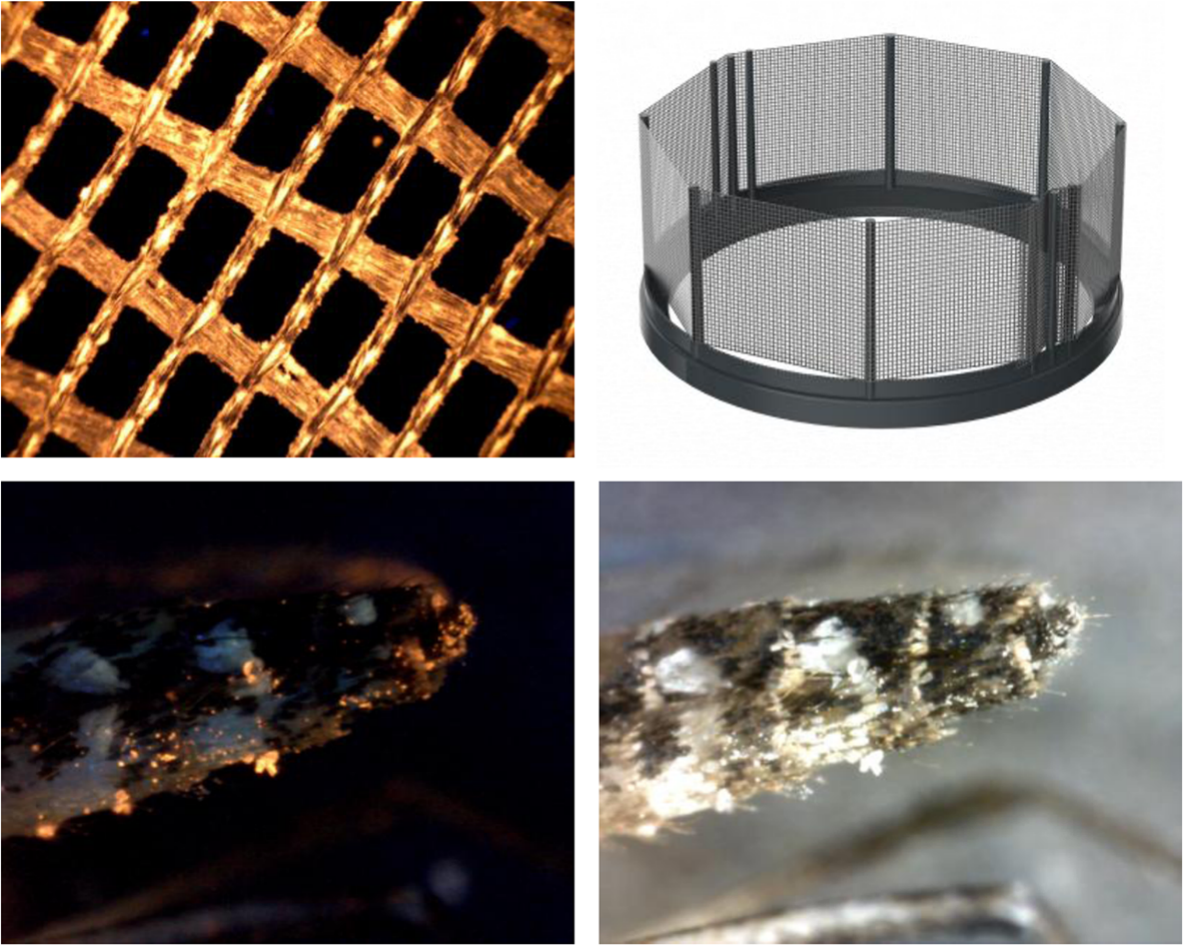
**Electrostatic gauze dusted with orange fluorescent powder under UV light (top left), floater-V with gauze (top right), the abdomen of an *Ae. aegypti *exposed to gauze with fluorescent powder (bottom left (UV) and bottom right).** Magnification 200x.

To maximize mosquito infection and contamination inside the trap, we evaluated and optimized the design of the floater component. We validated the attractiveness of the gauze-carrying floater by measuring mosquito contact using fluorescent dust. We tested different floater types and sizes (Table 2), in experimental cages where 50 gravid mosquitoes were released and retrieved after 2 days to observe the presence of fluorescent powder on the mosquitoes using a UV light microscope. Since only the gauze was dusted with fluorescent powder, the presence of this powder on mosquitoes was used as a measure for contact with the floater gauze and served as a proxy for the attractiveness and efficacy of the floater components. Gauze with fluorescent dust and mosquitoes with fluorescent particles are shown in Figure 5.

**Figure 6:**
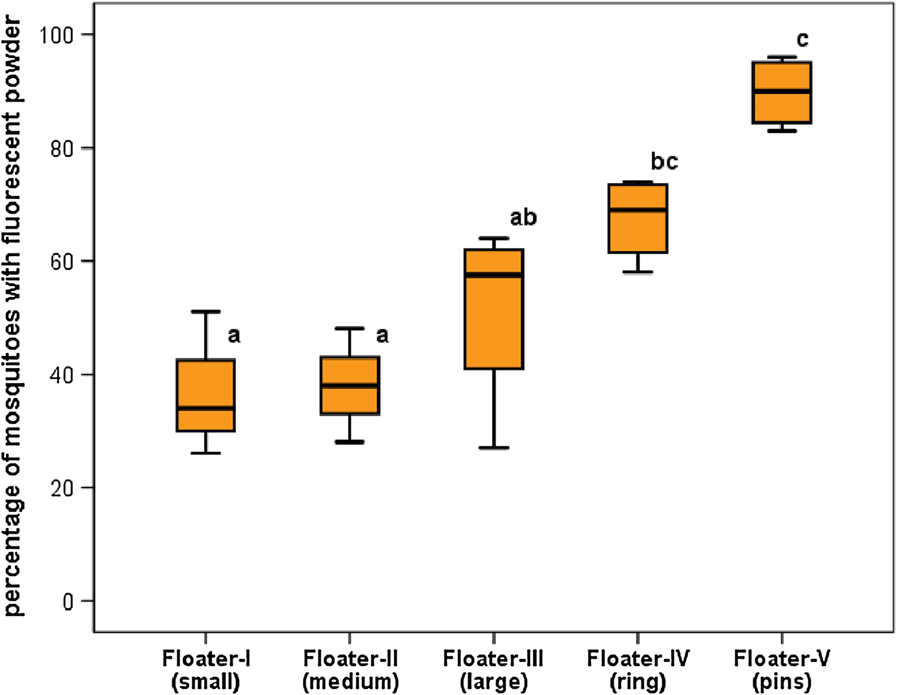
**Percentage (± SE) of retrieved mosquitoes with fluorescent powder.** Comparisons were made between 5 different floaters in prototype B (see Figure 1). n = 3 for Floater-I and Floater-II, n = 4 for Floater-III, Floater-IV and Floater-V. Each box denotes the median as a line across the middle and the quartiles (25th and 75th percentiles) at the bottom and top. Treatments without letters in common are significant different at P < 0.05.

Results showed high percentages of mosquitoes with fluorescent powder in particular for the floaters with a large gauze surface (Figure 6). Significant differences in the attractiveness and mosquito contact of the different floaters were observed. The percentage of mosquitoes with fluorescent powder was significantly higher for Floater-IV and V compared to Floater-I (p = 0.025 and p < 0.001, respectively) and Floater-II (p = 0.030 and p < 0.001, respectively). Floater-V was also more attractive than Floater-III (p = 0.003). Overall, the increase in gauze surface on the floaters increased the percentage of mosquitoes with fluorescent powder (Figure 6). This indicates that the floater provides effective contact and powder transfer to resting/ovipositing mosquitoes and can be used to contaminate these once inside the trap. In all experiments, we observed much higher numbers of mosquito eggs laid inside the prototype device compared to the alternative sites, which indicates that the In2Care®Mosquito Trap is more attractive to gravid *Ae. aegypti* than the open black flower pots.

The final floater design (Floater-V, Table 2) was effective in contaminating, on average, 90% of the retrieved mosquitoes. This design is based on a thin polyethylene ring that floats via five air-chambers in the bottom and has protruding pins onto which the gauze can be fixed (Figure 5). This design allows the use of control agents on both sides of the gauze and enables egg-laying mosquitoes to sit close to the water surface. We therefore selected this floater as the standard floater for the trap.

### Mosquitocidal agents

Multiple experiments were conducted to test and improve the impact of mosquitocidal agents in the trap. Gauze strips were dusted with a mixture of *Beauveria bassiana* spores and pyriproxyfen particles (as described in more detail in the Methods section) and applied inside the trap using Floater V.

### Impact of *Beauveria bassiana* on adult mosquitoes

**Figure 7:**
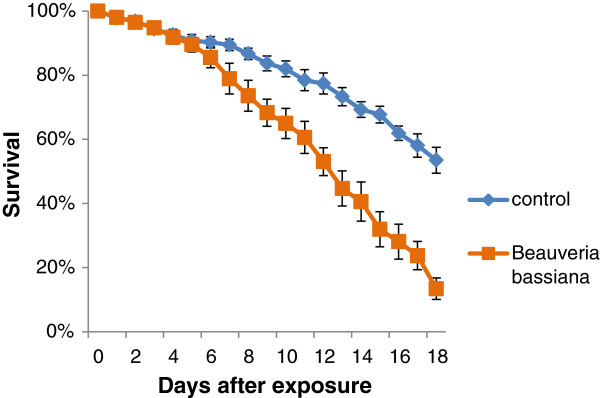
**Survival curves (± SE) of mosquitoes exposed to *Beauveria bassiana *(n = 8) deployed in the In2Care mosquito Trap, compared to a control group (n = 8).** Adult survival was monitored daily for 18 days after recapture. Survival curves were significantly different at P < 0.001.

Mosquitoes retrieved from cages with the trap showed a reduced survival compared to control groups. The Kaplan-Meier LT_50_ estimation of the mosquitoes infected with *Beauveria bassiana* spores was 14.00 days (13.21-14.80, 95% CI, Figure 7). The LT_50_ of control groups was beyond the 18 days measured so could not be calculated with the Kaplan-Meier model.

Survival curves were significantly different for mosquitoes infected with the fungus compared to controls (p < 0.001, Kaplan-Meier with logrank test). This impact on adult survival demonstrates that the *B. bassiana* spores applied on the floater gauze are effective in contaminating *Aedes* mosquitoes with high infection doses even with uncontrolled, realistic and potentially short exposure times (Figure 7). The relatively slow killing process of the fungus enables the contaminated females to spread pyriproxyfen to other breeding sites.

### Impact of pyriproxyfen on larvae

**Figure 8:**
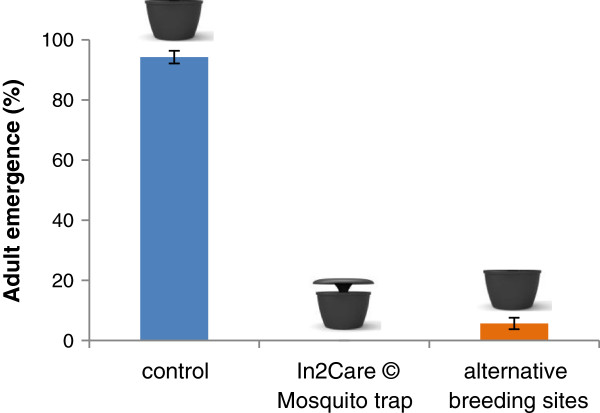
**Adult emergence (% ± SE) in dissemination experiments.** Emergence was significantly (P < 0.05) reduced in the In2Care Mosquito Trap and the alternative breeding sites (n = 4) compared to the control larvae (n = 4).

Results showed that pyriproxyfen was actively dispersed from the trap to the surrounding breeding sites via the contaminated mosquitoes; killing on average >90% of all developing larvae in these sites. This autodissemination impact significantly reduced the emergence of adult mosquitoes in the breeding sites around the device (less than 1 in 10 larvae survived to adulthood) compared to control larvae of which ca. 95% larvae developed into adults (Figure 8, p < 0.001, one-way ANOVA test). Furthermore, we found a significant reduction in larval survival inside the trap compared to the control larvae (p < 0.001, one-way ANOVA test). We consistently observed 100% mortality of the larvae in the trap due to the presence of pyriproxyfen in the water of the trap. It is noteworthy that no additional pyriproxyfen was added to the water in the trap or the alternative breeding sites, which demonstrates that the amount that is transferred into the water solely via visiting ovipositing mosquitoes is sufficient to effectively kill larvae.

## Discussion

The In2Care® Mosquito Trap was designed to provide a novel tool to control *Ae. aegypti* mosquitoes. The trap has the advantage of operating without the need for electricity or carbon dioxide and is made from low-cost polyethylene, which makes it a relatively cheap tool for mosquito control. The trap design is suitable for high throughput manufacturing and utilizes commercially available ingredients.

Results showed that this yeast-baited water-filled ovitrap can effectively attract gravid *Ae. aegypti*. Furthermore, the use of pyriproxyfen is expected to further increase mosquito attraction. Because pyriproxyfen is a late-stage killing agent, targeting the pupal stages only, the deposited eggs inside the device will still hatch and develop into larvae which results in the accumulation of larval odors in the trap over time. Studies show that volatiles emitting from larvae are attractive to gravid Dengue vectors [12], and can therefore be expected to augment the attractiveness of the trap.

While most vector control tools focus on either adults or larvae, the In2Care® Mosquito Trap targets both larval and adult life stages of *Ae. aegypti*. Results showed that the powders applied in the trap exert effective mortality impacts on contaminated adult mosquitoes, in the larvae developing inside the trap, as well as the on larval development in surrounding breeding sites. Results also showed that gravid females pick up lethal doses of control agents upon short and transient contact with the floater gauze. The use of fungal spores was selected as an environmentally-friendly alternative to chemical pesticides. The relatively slow mode of action of the fungus *B. bassiana* provides a long-lasting adult control option by targeting only the older females that can transmit disease, thereby drastically reducing the chances for development of resistance [35]. Another major advantage of this fungus is that it causes a reduction in Dengue virus transmission via interference with virus replication inside the mosquito [30]. Other pre-lethal transmission-blocking effects observed in fungus-infected adults include a reduced fecundity and blood feeding propensity, which causes a significant reduction in their vectorial capacity. Moreover, the use of *B. bassiana* in the trap allowed the use of the autodissemination agent pyriproxyfen. It was found that pyriproxyfen was highly effective when deployed inside the trap, because in all experiments 100% of the larvae in the trap died at the time of pupation. Furthermore, we found a significant reduction in larval survival in the breeding sites surrounding the trap compared to the control larvae. This proves that pyriproxyfen was effectively dispersed to other breeding sites by free-flying mosquitoes in the experimental cages, which shows the possibility of controlling *Aedes* mosquitoes using the autodissemination effect, previously described by Devine *et al. *[25] and Caputo *et al. *[22]. Because pyriproxyfen does not have a repellent effect or impact on adult mortality, it allows the full exploitation of the skip-oviposition behavior of *Aedes* mosquitoes. Particularly in areas where breeding sites are abundant and transient during the wet season, the use of pyriproxyfen provides an exciting opportunity for precision-targeted larval control using the female mosquito itself.

Potentially, other insecticidal agents could also be used in the device, such as fast-killing chemicals like the carbamate bendiocarb. Investigations on the efficacy of the trap using these and other insecticidal agents are currently ongoing. Whereas most vector control chemicals will have the advantage of a fast killing effect (and thereby the visual confirmation of dead mosquitoes inside the trap), this effect will not be useful when using autodissemination agents, which require the contaminated mosquito to survive for at least a few days to successfully spread pyriproxyfen to surrounding breeding sites.

Although the trap was originally designed to lure *Ae. aegypti* mosquitoes, we have seen *Culex* species resting inside the trap during field trails. These observations show good potential for further investigations on the attractiveness of the trap for other mosquito species (for example other culicines and maybe even anophelines). It has been shown that multiple disease transmitting mosquito species can be targeted using ovitraps, such as *Ae. albopictus *[7,15,16], *Cx. quinquefasciatus *[16,17,36] and *Cx. pipiens *[37]. In future studies, we will evaluate additional lures to attract other species, which could improve pyriproxyfen dissemination coverage and broader uptake of this control tool. Large-scale field tests with epidemiological outputs are the next logical step in this research. Additional field validations will be needed to further examine and augment the attractiveness of the trap in a more natural situation, and to gain more knowledge on the optimal placement and deployment of the traps in a variety of settings.

## Conclusions

The trap described in this work can have an advantage over existing control tools in integrated vector control strategies aimed at significantly reducing *Ae. aegypti* populations, because of the unique multi-dimensional effect of trap. Like other ovitraps, the trap does not provide personal protection, which means that additional measures to prevent disease transmission will be needed. However, because the trap also has an effect on breeding sites in the vicinity of the trap it could be a useful tool in integrated vector management campaigns to provide protection around the house and in public places. Large-scale deployment and proper placement and servicing of the trap will be needed to be able to reduce Dengue vector densities and disease transmission. Trap servicing will be needed every 6-8 weeks, to refill the trap with water, add an odor tablet and to replace the gauze with control agents. Even with this frequent need for servicing, the trap’s low cost price makes it a promising addition to current Dengue vector control tools.

## Competing interests

All authors are remunerated and/or employed by In2Care BV and have commercial stakes with regard to the device described in this article.

## Authors’ contributions

JS and RA contributed to the experimental designs, conducted laboratory experiments, performed the data analysis and JS drafted the manuscript. RAS contributed to the experimental designs and AJO contributed with device developments and improvements. BGJK and MF designed the experiments and revised the manuscript. All authors read and approved the final version of the manuscript.

## References

[B1] Dengue and severe dengue, fact sheet N°117 http://www.who.int/mediacentre/factsheets/fs117/en/index.html

[B2] Bhatt S Gething PW Brady OJ Messina JP Farlow AW Moyes CL Drake JM Brownstein JS Hoen AG Sankoh O Myers MF George DB Jaenisch T Wint WGR Simmons CP Scott TW Farrar JJ Hay SI The global distribution and burden of dengue Nature 2013 496 504 507 10.1038/nature12060 23563266 PMC3651993

[B3] Harrington J Kroeger A Runge-Ranzinger S O’Dempsey T Detecting and responding to a dengue outbreak: evaluation of existing strategies in country outbreak response planning J Trop Med 2013 2013 756832 24222774 10.1155/2013/756832 PMC3810135

[B4] Reiter P Oviposition, dispersal, and survival in aedes aegypti: implications for the efficacy of control strategies Vector Borne Zoonotic Dis 2007 7 261 273 10.1089/vbz.2006.0630 17627447

[B5] Maciel-de-Freitas R Lourenco-de-Oliveira R Does targeting key-containers effectively reduce aedes aegypti population density? Tropical Medicine Int Health: TM & IH 2011 16 965 973 10.1111/j.1365-3156.2011.02797.x 21605290

[B6] Fay RW Elison DA A preferred oviposition site as a surveillance method for aedes aegypti Mosq news 1966 26 531 535

[B7] Facchinelli L Valerio L Pombi M Reiter P Costantini C Della Torre A Development of a novel sticky trap for container-breeding mosquitoes and evaluation of its sampling properties to monitor urban populations of aedes albopictus Med Vet Entomol 2007 21 183 195 10.1111/j.1365-2915.2007.00680.x 17550438

[B8] Chadee DD Ritchie SA Efficacy of sticky and standard ovitraps for aedes aegypti in Trinidad, West Indies J Vector Ecol: journal of the Society for Vector Ecology 2010 35 395 400 10.1111/j.1948-7134.2010.00098.x 21175947

[B9] Santana AL Roque RA Eiras AE Characteristics of grass infusions as oviposition attractants to aedes (Stegomyia) (Diptera: Culicidae) J Med Entomol 2006 43 214 220 10.1603/0022-2585(2006)043[0214:COGIAO]2.0.CO;2 16619601

[B10] Ritchie SA Effect of some animal feeds and oviposition substrates on aedes oviposition in ovitraps in Cairns, Australia J Am Mosq Control Assoc 2001 17 206 208 14529089

[B11] Ponnusamy L Xu N Nojima S Wesson DM Schal C Apperson CS Identification of bacteria and bacteria-associated chemical cues that mediate oviposition site preferences by aedes aegypti Proc Natl Acad Sci USA 2008 105 9262 9267 10.1073/pnas.0802505105 18607006 PMC2443818

[B12] Wong J Stoddard ST Astete H Morrison AC Scott TW Oviposition site selection by the dengue vector aedes aegypti and its implications for dengue control PLoS Negl Trop Dis 2011 5 e1015 10.1371/journal.pntd.0001015 21532736 PMC3075222

[B13] Eiras AE Resende MC Preliminary evaluation of the ‘dengue-MI’ technology for aedes aegypti monitoring and control Cad Saude Publica 2009 25 Suppl 1 S45 S58 19287866 10.1590/s0102-311x2009001300005

[B14] Resende MC Azara TM Costa IO Heringer LC Andrade MR Acebal JL Eiras AE Field optimisation of MosquiTRAP sampling for monitoring aedes aegypti Linnaeus (Diptera: culicidae) Mem Inst Oswaldo Cruz 2012 107 294 302 10.1590/S0074-02762012000300002 22510823

[B15] Gama RA Silva EM Silva IM Resende MC Eiras AE Evaluation of the sticky MosquiTRAP for detecting aedes (stegomyia) aegypti (L.) (Diptera: culicidae) during the dry season in Belo Horizonte, Minas Gerais, Brazil Neotrop Entomol 2007 36 294 302 10.1590/S1519-566X2007000200018 17607465

[B16] de Santos EM de Melo-Santos MA de Oliveira CM Correia JC de Albuquerque CM Evaluation of a sticky trap (AedesTraP), made from disposable plastic bottles, as a monitoring tool for aedes aegypti populations Parasit Vectors 2012 5 195 10.1186/1756-3305-5-195 22958376 PMC3464176

[B17] Ritchie SA Long S Hart A Webb CE Russell RC An adulticidal sticky ovitrap for sampling container-breeding mosquitoes J Am Mosq Control Assoc 2003 19 235 242 14524545

[B18] Williams CR Ritchie SA Long SA Dennison N Russell RC Impact of a bifenthrin-treated lethal ovitrap on aedes aegypti oviposition and mortality in north Queensland, Australia J Med Entomol 2007 44 256 262 10.1603/0022-2585(2007)44[256:IOABLO]2.0.CO;2 17427694

[B19] Zeichner BC Perich MJ Laboratory testing of a lethal ovitrap for aedes aegypti Med Vet Entomol 1999 13 234 238 10.1046/j.1365-2915.1999.00192.x 10514047

[B20] Ritchie SA Long SA McCaffrey N Key C Lonergan G Williams CR A biodegradable lethal ovitrap for control of container-breeding aedes J Am Mosq Control Assoc 2008 24 47 53 10.2987/5658.1 18437814

[B21] Hemingway J Ranson H Insecticide resistance in insect vectors of human disease Annu Rev Entomol 2000 45 371 391 10.1146/annurev.ento.45.1.371 10761582

[B22] Caputo B Ienco A Cianci D Pombi M Petrarca V Baseggio A Devine GJ Della Torre A The “auto-dissemination” approach: a novel concept to fight aedes albopictus in urban areas PLoS Negl Trop Dis 2012 6 e1793 10.1371/journal.pntd.0001793 22953015 PMC3429402

[B23] Itoh T Kawada H Abe A Eshita Y Rongsriyam Y Igarashi A Utilization of bloodfed females of aedes aegypti as a vehicle for the transfer of the insect growth regulator pyriproxyfen to larval habitats J Am Mosq Control Assoc 1994 10 344 347 7807075

[B24] Ohba S-y Ohashi K Pujiyati E Higa Y Kawada H Mito N Takagi M The effect of pyriproxyfen as a “population growth regulator” against aedes albopictus under semi-field conditions PloS One 2013 8 e67045 10.1371/journal.pone.0067045 23843982 PMC3699564

[B25] Devine GJ Perea EZ Killeen GF Stancil JD Clark SJ Morrison AC Using adult mosquitoes to transfer insecticides to aedes aegypti larval habitats Proc Natl Acad Sci U S A 2009 106 11530 11534 10.1073/pnas.0901369106 19561295 PMC2702255

[B26] Ponlawat A Fansiri T Kurusarttra S Pongsiri A McCardle PW Evans BP Richardson JH Development and evaluation of a pyriproxyfen-treated device to control the dengue vector, aedes aegypti (L.) (Diptera:culicidae) Southeast Asian J Trop Med Public Health 2013 44 167 178 23691625

[B27] Farenhorst M Mouatcho JC Kikankie CK Brooke BD Hunt RH Thomas MB Koekemoer LL Knols BG Coetzee M Fungal infection counters insecticide resistance in African malaria mosquitoes Proc Natl Acad Sci U S A 2009 106 17443 17447 10.1073/pnas.0908530106 19805146 PMC2762667

[B28] Blanford S Shi W Christian R Marden JH Koekemoer LL Brooke BD Coetzee M Read AF Thomas MB Lethal and pre-lethal effects of a fungal biopesticide contribute to substantial and rapid control of malaria vectors PloS ONE 2011 6 e23591 10.1371/journal.pone.0023591 21897846 PMC3163643

[B29] Darbro JM Johnson PH Thomas MB Ritchie SA Kay BH Ryan PA Effects of beauveria bassiana on survival, blood-feeding success, and fecundity of aedes aegypti in laboratory and semi-field conditions Am J Trop Med Hyg 2012 86 656 664 10.4269/ajtmh.2012.11-0455 22492151 PMC3403760

[B30] Dong Y Morton JC Jr Ramirez JL Souza-Neto JA Dimopoulos G The entomopathogenic fungus beauveria bassiana activate toll and JAK-STAT pathway-controlled effector genes and anti-dengue activity in aedes aegypti Insect Biochem Mol Biol 2012 42 126 132 10.1016/j.ibmb.2011.11.005 22198333 PMC3462650

[B31] Kikankie CK Brooke BD Knols BG Koekemoer LL Farenhorst M Hunt RH Thomas MB Coetzee M The infectivity of the entomopathogenic fungus beauveria bassiana to insecticide-resistant and susceptible anopheles arabiensis mosquitoes at two different temperatures Malar J 2010 9 71 10.1186/1475-2875-9-71 20210990 PMC2839986

[B32] Farenhorst M Knols BG Thomas MB Howard AF Takken W Rowland M N’Guessan R Synergy in efficacy of fungal entomopathogens and permethrin against west African insecticide-resistant anopheles gambiae mosquitoes PloS One 2010 5 e12081 10.1371/journal.pone.0012081 20711409 PMC2920335

[B33] Ponnusamy L Xu N Boroczky K Wesson DM Abu Ayyash L Schal C Apperson CS Oviposition responses of the mosquitoes aedes aegypti and aedes albopictus to experimental plant infusions in laboratory bioassays J Chem Ecol 2010 36 709 719 10.1007/s10886-010-9806-2 20521087 PMC4562425

[B34] Bukhari T Knols BG Efficacy of aquatain, a monomolecular surface film, against the malaria vectors anopheles stephensi and an: gambiae s.s in the laboratory Am J Trop Med Hyg 2009 80 758 763 19407120

[B35] Read AF Lynch PA Thomas MB How to make evolution-proof insecticides for malaria control PLoS Biol 2009 7 e1000058 19355786 10.1371/journal.pbio.1000058 PMC3279047

[B36] Barbosa RM Souto A Eiras AE Regis L Laboratory and field evaluation of an oviposition trap for culex quinquefasciatus (Diptera: culicidae) Mem Inst Oswaldo Cruz 2007 102 523 529 10.1590/S0074-02762007005000058 17612774

[B37] Jackson BT Paulson SL Youngman RR Scheffel SL Hawkins B Oviposition preferences of culex restuans and culex pipiens (Diptera: culicidae) for selected infusions in oviposition traps and gravid traps J Am Mosq Control Assoc 2005 21 360 365 10.2987/8756-971X(2006)21[360:OPOCRA]2.0.CO;2 16506560

